# Utilization Trends of Glucose-Lowering Medications Among Adult Kidney Transplant Recipients with Type 2 Diabetes in the United States

**DOI:** 10.3390/jcm14020651

**Published:** 2025-01-20

**Authors:** Panupong Hansrivijit, Helen Tesfaye, Deborah J. Wexler, Reza Abdi, Elisabetta Patorno, Julie M. Paik

**Affiliations:** 1 Division of Pharmacoepidemiology & Pharmacoeconomics, Brigham and Women’s Hospital, Boston, MA 02115, USA; htesfaye@mgb.org (H.T.); epatorno@bwh.harvard.edu (E.P.); jmpaik@bwh.harvard.edu (J.M.P.); 2 Division of Renal (Kidney) Medicine, Brigham and Women’s Hospital, Boston, MA 02115, USA; rabdi@bwh.harvard.edu; 3 Harvard Medical School, Boston, MA 02115, USA; dwexler@mgh.harvard.edu; 4 Diabetes Center, Massachusetts General Hospital, Boston, MA 02114, USA; 5 Transplantation Research Center, Brigham and Women’s Hospital, Boston, MA 02115, USA

**Keywords:** diabetes, kidney transplant, prescribing trends, SGLT2 inhibitors, GLP-1 receptor agonists

## Abstract

**Background:** To date, there are limited studies describing the use of glucose-lowering medications (GLMs) in adult kidney transplant recipients (KTRs), and the uptake of sodium glucose cotransporter-2 inhibitors (SGLT2is) and glucagon-like peptide-1 receptor agonists (GLP1RAs). Thus, we aimed to evaluate the use of GLMs, including SGLT2i and GLP1RA, among adult KTRs with type 2 diabetes (T2D). **Methods:** This is an ecologic study of adult KTR with T2D. Data were sourced from two large U.S. health insurance claim databases from 2014 to 2023. The proportions of any user and incident use of GLMs were reported in percentage. Any use of GLM was defined through prescription claims, and incident use was further defined as the absence of any prior dispensing within the preceding 365 days. **Results:** From 2014 to 2023, we identified 33,913 adult KTRs with T2D who were prescribed any GLMs. Any use of SGLT2i and GLP1RA increased throughout the study period (0.4% to 14.4% for SGLT2i, and 2.8% to 12.5% for GLP1RA). While insulin was the most frequently used GLM, ranging from 58% to 74%, the usage gradually declined over time. By 2023, SGLT2i and GLP1RA were initiated nearly as frequently as insulin (5.1% for SGLT2i, 5.7% for GLP1RA, and 5.7% for insulin). Compared with insulin initiators, SGLT2i initiators (*n* = 1009) had a higher prevalence of cardiovascular comorbidities and proteinuria, while GLP1RA initiators (*n* = 2149) had a higher prevalence of obesity. **Conclusions:** Any use of both SGLT2i and GLP1RA among KTRs with T2D increased over time with the incident use of SGLT2i and GLP1RA as high as insulin by 2023. Our findings emphasize the need for the effectiveness and safety analysis of SGLT2i and GLP1RA among KTRs with T2D.

## 1. Introduction

Type 2 diabetes (T2D) is a common cause of end-stage kidney disease [1] and accounts for approximately 60% of all kidney transplants in the United States [2]. In clinical practice, dysglycemia is very common after kidney transplantation. Maintaining good glycemic control is crucial for kidney transplant recipients (KTRs) with T2D, as poor glycemic control after kidney transplantation is associated with adverse graft and patient outcomes [3].

Among the various glucose-lowering medications (GLMs) currently available, insulin is generally considered the first-line therapy for KTRs with T2D, especially for patients with varying degrees of kidney impairment [4]. Other traditional agents, such as biguanides (i.e., metformin), sulfonylureas (SU), and thiazolidinediones (TZD), may also be used in KTRs with T2D [4]. However, there are no consensus practice guidelines on GLM selection for KTRs with T2D.

Newer GLMs, especially sodium glucose cotransporter-2 inhibitors (SGLT2is) and glucagon-like peptide-1 receptor agonists (GLP1RAs), have been increasingly prescribed to non-transplant patients with chronic kidney disease (CKD), with or without T2D [5]. This practice trend is based on the findings from several randomized controlled trials (RCTs) that consistently reported cardiovascular (CV) and renal benefits with SGLT2i and GLP1RA therapy [6,7,8,9]. Lentine et al. reported that the fill rate of SGLT2i has a rising trend [10]. However, the fill rate reported did not differentiate incident use from any use of the medications. Moreover, the utilization trends of GLP1RA were not reported. To date, the utilization trends of SGLT2i and GLP1RA among KTRs with T2D using these medications needs further exploration.

Hence, in this study, we aim to evaluate the utilization trends of GLMs, including SGLT2i and GLP1RA, from 2014 to 2023, as well as the clinical characteristics of KTR with treated T2D, using two large U.S. commercial insurance claim databases.

## 2. Materials and Methods

### 2.1. Data Sources

We used data from two large U.S. commercial health insurance claim databases: Optum’s de-identified Clinformatics^®^ Data Mart (referred to as Clinformatics^®^ hereafter), from 1 January 2014, to 30 November 2023) and IBM MarketScan^®,^ Armonk, NY, USA, from 1 January 2014 to 31 December 2021. These de-identified datasets are comprised of commercial health plan members and Medicare Advantage members across all 50 states and the District of Columbia. Longitudinal information on clinical characteristics, procedures, inpatient and outpatient diagnoses, and outpatient prescription dispensing is available for all enrollees. Laboratory results are available for a subset of patients (40% from Clinformatics^®^, Eden Prairie, MN, USA, and 5–10% from IBM MarketScan^®^, Armonk, NY, USA). This study was approved by the Mass General Brigham Institutional Review Board, and data use agreements were in place.

### 2.2. Study Design

This is an ecologic study delineating the utilization trends of GLMs and the clinical characteristics of KTRs with T2D who were treated with at least one GLM from 2014 to 2023. We adopted both any user and incident user study designs.

### 2.3. Study Population

We included adult KTRs with a concurrent history of T2D who were treated with at least one GLM (heretofore referred to as “KTR with treated T2D”) from 1 January 2014, to the most recent available data (30 November 2023 for Clinformatics^®^ and 31 December 2021 for IBM MarketScan^®^). Kidney transplant recipients were identified by International Classification of Diseases (ICD) codes V42.xx, and Z94.xx [11]. We selected 1 January 2014, as the start of the study period because the most recent class of GLMs, SGLT2i, was approved by the United States Food and Drug Administration in the second half of 2013. The cohort entry date was the first date that patients were prescribed at least one GLM during 1 January 2014 to 30 November 2023. We excluded patients aged ≤ 18 years, and patients with <365 days of continuous enrollment in an insurance plan prior to the cohort entry date.

### 2.4. Any Users and Incident Users of Glucose-Lowering Medications

Any users were defined as patients who had prescription claims for any given GLM from 1 January 2014 to 30 November 2023, irrespective of prior use history. Incident users were defined as new initiators of a given GLM without prior use of that medication class within 365 days. GLMs were grouped into classes; the assessed GLM classes included SGLT2i, GLP1RA, insulins, metformin, second-generation SU, dipeptidyl peptidase-4 inhibitors (DPP4i), TZD, and miscellaneous agents (first-generation SU, α-glucosidase inhibitors, pramlintide, and meglitinide).

### 2.5. Clinical Characteristics

We investigated the clinical characteristics of all KTRs with treated T2D (defined as any use of GLMs) from the overall study period (1 January 2014 to 30 November 2023). We also investigated the clinical characteristics of patients who were newly initiated on SGLT2i, GLP1RA, or insulin.

The clinical characteristics were measured during the 365 days prior to the cohort entry date. These included demographics, comorbidity score [12], frailty score [13], diabetes complications, cardiovascular, renal and other comorbidities, and other medications [5]. Obesity was defined by ICD diagnosis codes for a body mass index (BMI) greater than 30.0 kg/m^2^. Overweight was defined by ICD diagnosis codes for a 25.0–29.9 kg/m^2^ BMI.

### 2.6. Statistical Analyses

Both any use and incident use of each GLM class are reported as a percentage, calculated by dividing the number of patients using each specific medication class by the total sample size (total number of patients treated with at least one GLM). To establish GLM utilization trends, we stratified the entire study period (1 January 2014 to 30 November 2023) into 6-month intervals. Any use and incident use of each GLM were calculated for each interval as the number of any users and incident users divided by the number of patients contributing at least one day of follow-up within that interval. The proportions of GLM class use from all intervals were plotted to create chronological trends across the study period. The trends from the 2022–2023 period were derived from Clinformatics^®^ data only due to data availability. We used the Mann–Kendall trend test to evaluate the presence of monotonic trends in the utilization rates of SGLT2i and GLP1RA over a study period from 2014 to 2023. For clinical characteristics, binary and categorical variables are reported as numbers and percentages, with the total sample size as the denominator. Continuous variables are reported as the means ± standard deviation (SD). Characteristics were compared between SGLT2i, GLP1RA, and insulin incident users using Chi-square (for categorical variables) and ANOVA tests (for continuous variables). All analyses were performed using Aetion^®^ Evidence Platform version 4.92.0 (Aetion, Inc., New York, NY, USA) and R (version 2023.06.0+421, Posit Software, PBC).

## 3. Results

### 3.1. Glucose-Lowering Medications

We identified 33,913 KTRs with treated T2D who were taking any GLMs (18,419 patients from Clinformatics^®^ and 15,494 from IBM MarketScan^®^) from 1 January 2014 to 30 November 2023 (Table 1). The mean number of GLMs per patient was 1.2 ± 0.5 medications (Table S1). Among KTRs with T2D taking any GLMs, most patients were prescribed insulin (75%), followed by second-generation SU (21.9%), metformin (21.8%), DPP4i (16.1%), GLP1RA (11.5%), SGLT2i (8.1%), and TZD (3.9%).

### 3.2. Trends of GLM Any Users, 2014–2023

**Figure 1:**
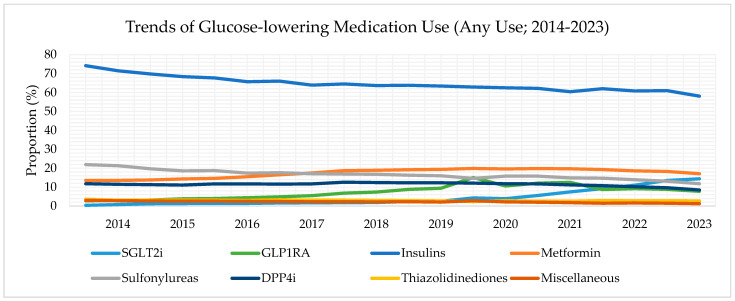
Trends of glucose-lowering medication use (any use) from pooled databases. Data range from 1 January 2014 to 30 November 2023. Note that the results after 31 December 2021 are attributed only to the Clinformatics^®^ database (shadowed area).

Among KTRs with T2D taking any GLMs, most patients were insulin users, but any users of insulin gradually declined over time (74.2% in the first half of 2014 to 58.1% in the latter half of 2023) (Figure 1). The proportion of any users of metformin increased from 13.6% in 2014 to 17.1% in 2023. The proportion of any users of sulfonylureas also decreased over time, from 21.9% in 2014 to 11.8% in 2023. The proportion of any users of DPP4i decreased from 11.8% in 2014 to 8.5% in 2023. The proportion of any users of TZD remained consistently low (<4%) from 2014 to 2023.

Any users of SGLT2i and GLP1RA increased over time (Figure 1). Among any users of GLMs, the proportion of any users of SGLT2i increased from 0.4% in the first half of 2014 to 14.4% in the latter half of 2023. The proportion of any users of GLP1RA also rose from 2.8% in the first half of 2014 to 7.8% in the latter half of 2023. For SGLT2i and GLP1RA any users, the Mann–Kendall test showed a significant upward trend (tau = 187; *p* < 0.001 and tau = 131; *p* < 0.001, respectively).

### 3.3. Trends of GLM Incident Users, 2014–2023

**Figure 2:**
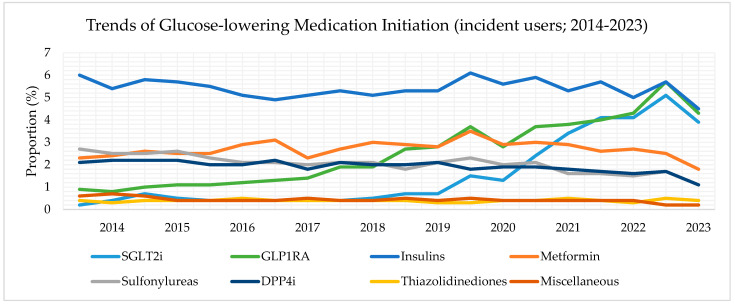
Trends of glucose-lowering medication initiation (incident use) from pooled databases. Data range from 1 January 2014 to 30 November 2023. Note that the results after 31 December 2021 are attributed only to the Clinformatics^®^ database (shadowed area).

Among KTRs with T2D initiating a GLM, the proportion of incident users of insulin was the highest across all GLMs. However, incident use of insulin gradually declined over time (6.0% in the first half of 2014 to 4.5% in the latter half of 2023) (Figure 2). We also observed decreases in the initiation of metformin, SU, DPP4i, and TZD throughout the study period. The proportion of incident users of metformin decreased from 2.3% in 2014 to 1.8% in 2023. Similarly, the proportion of incident users of SU decreased from 2.7% in 2014 to 1.1% in 2023. For DPP4i, the proportion of incident users declined from 2.1% in 2014 to 1.1% in 2023. The proportion of TZD incident users remained persistently low (<1%) throughout the study period.

The proportion of incident users of SGLT2i and GLP1RA increased over time. SGLT2i initiation rose sharply after 2019, and GLP1RA initiation gradually increased after 2017 (Figure 2). In the first half of 2014, the proportion of KTRs with treated T2D initiated on SGLT2i and GLP1RA was 0.2% and 0.9%, respectively, whereas, in the latter half of 2023, 3.9% of KTRs with treated T2D were initiated on SGLT2i and 4.3% were initiated on GLP1RA. For SGLT2i and GLP1RA incident users, the Mann–Kendall test showed a significant upward trend (tau = 138; *p* < 0.001 and tau = 179; *p* < 0.001, respectively).

### 3.4. Clinical Characteristics of KTRs with Treated T2D

Among all 33,913 patients, the mean age of KTRs with T2D taking any GLMs was 59.3 ± 11.0 years, with a majority being male (62.1%) and of white race (48.6%) (Table 1). The mean HbA1c was 7.4 ± 1.6%. The most prevalent diabetic complications were neuropathy (35.5%) and diabetic retinopathy (31.5%). Hyperglycemic crises, such as diabetic ketoacidosis and hyperosmolar hyperglycemic nonketotic syndrome, were reported in less than 5% of patients, while history of hypoglycemia was reported in 10.3% over the study period (Table 1).

A substantial proportion of KTRs with treated T2D had CV comorbidities. Throughout the study period, 91.1% had hypertension and 76.7% had hyperlipidemia. Coronary atherosclerosis and congestive heart failure were present in 34.3% and 24.7% of patients, respectively. Atrial fibrillation was reported in 13.5% of patients, and 12.0% had a history of ischemic stroke. Moreover, about 32.4% of all patients had codes for obesity and 9.7% had codes for being overweight. The annual prevalence of obesity was 37.2% in 2021, while the annual prevalence of overweight was 12.3% in 2021.

Of the 33,913 KTRs with treated T2D in the cohort, 1009 were initiated on SGLT2i, 2149 were initiated on GLP1RA, and 13,641 were initiated on insulin (Table 1 and Table S1). Compared with insulin initiators, patients who were initiated on SGLT2i were slightly older, more likely to be of black race, had fewer diabetic complications (ketoacidosis, neuropathy, retinopathy, hypoglycemia), more cardiovascular comorbidities (such as hypertension, congestive heart failure, atrial fibrillation, and ischemic stroke), more proteinuria and history of genital mycotic infection, and were less likely to have history of a urinary tract infection (UTI), hyperkalemia, and acute kidney injury (AKI). Compared with patients who initiated insulin, patients who were initiated on SGLT2i had higher use of renin–angiotensin system inhibitors, digoxin, anti-anginal agents, anti-arrhythmics, statins, and anti-coagulants, but lower the use of loop diuretics and potassium binders.

Compared with patients who were initiated on insulin, patients who were initiated on GLP1RA were younger, had a lower comorbidity score, frailty score, and diabetic complications, higher prevalence of obesity, and history of genital mycotic infection. The prevalence of coronary atherosclerosis, congestive heart failure, atrial fibrillation, ischemic stroke, and peripheral arterial disease was lower in GLP1RA initiators compared with insulin initiators. The use of statins, beta-blockers, antiplatelet agents, and anticoagulants was lower in GLP1RA initiators compared with insulin initiators.

## 4. Discussion

Among KTRs with treated T2D, there was an overall increase in SGLT2i utilization (both any use and incident use), accompanied by a decline in other GLMs, such as insulin, metformin, and SU over the study period. The initiation of SGLT2i noticeably started after 2019 and was initiated almost as frequently as insulin by 2023. Our study also found that GLP1RA initiation among KTRs with T2D gradually increased over time. The proportion of patients who were initiated on a GLP1RA was almost as high as the proportion of patients initiated on insulin by the end of our study period. Compared with patients initiated on insulin, patients initiated on SGLT2i had a higher prevalence of CV comorbidities and proteinuria, whereas patients initiated on GLP1RA had a higher prevalence of obesity.

Although KTRs with treated T2D were excluded from RCTs of SGLT2i [6,7,8], the rise in SGLT2i utilization in recent years suggests that clinicians are nonetheless prescribing these medications to KTRs with T2D. Our finding of rising SGLT2i initiation in contrast with declining insulin initiation suggests that prescribers are shifting to prescribing SGLT2i over insulin, especially for KTRs who have a high burden of CV disease and proteinuria to optimize CV [6,7,8] and renal benefits [14,15,16]. Given the utilization trends in our study, we anticipate that SGLT2i will be increasingly prescribed to KTRs with T2D as clinicians become more familiar with this medication class.

Our finding of a lower prevalence of UTIs among SGLT2i initiators could indicate that clinicians tended to avoid prescribing SGLT2i for KTRs with a history of UTI, despite growing evidence that SGLT2is are well-tolerated among non-transplant patients with CKD and T2D. A population-based study of patients with CKD and T2D found that SGLT2i use was associated with an increased risk of genital infection but not severe UTIs [14,17]. One retrospective cohort study of 57 diabetic KTRs reported that SGLT2i was not associated with an increased risk of genital infection and UTI [18]. However, this study was limited by the very small sample size and thus may not have been sufficiently powered to detect these outcomes. In our study, compared with insulin initiators, it was notable that the prevalence of a history of genital mycotic infection was higher not only in patients who were initiated on SGLT2i, but also in patients who were initiated on GLP1RA. Given that patients who were initiated on GLP1RA were more obese, it is possible that obesity could contribute to an increased risk of genital mycotic infection in KTRs with T2D. Additional investigations are needed to delineate the risk of UTI and genital mycotic infection associated with SGLT2i among KTRs with T2D.

Our finding of a lower prevalence of AKI and hyperkalemia among SGLT2i initiators could indicate that SGLT2is were less likely to be prescribed in patients with a prior history of AKI and/or hyperkalemia. Interestingly, among non-transplant CKD patients with T2D, SGLT2i use has been associated with a lower risk of AKI [18,19,20] and reduced the risk of hyperkalemia [20]. Nonetheless, the risk of AKI and hyperkalemia among KTRs with T2D remains unknown and should be investigated in future studies.

GLP1RAs have been used for glycemic control and weight loss, regardless of diabetes status [21]. As such, we consistently found that KTRs with treated T2D who were initiated on a GLP1RA had a higher prevalence of obesity. The rising trend of GLP1RA initiation corresponded with the release of a series of GLP1RA landmark trials from 2015 to 2021 [22]. However, none of these trials included patients with a history of kidney transplantation. Thus, the upward trend of GLP1RA initiation in our study suggests that clinicians are prescribing GLP1RA more frequently to KTRs with T2D, especially when their patients have obesity. Furthermore, we found that the proportion of patients who were initiated on GLP1RA was on par with those who were initiated on insulin, which suggests that clinicians may be considering patients’ comorbidities when deciding among the GLMs to maximize the effect on glycemic control, as well as metabolic complications, such as weight loss [23,24,25,26,27,28]. However, at this time, there are no studies on the effectiveness and safety of GLP1RA among KTRs with T2D.

Our study has limitations. Firstly, our study serves as a pilot study for comparative effectiveness and safety analysis. Thus, it is worth noting that SGLT2is or GLP1RAs were prescribed for either glycemic control, cardio-renal protection, or weight loss, respectively. The indications of these medications will be delineated in the comparative effectiveness and safety analysis. Secondly, although our two databases covered Medicare Advantage patients, our patient population are different from primary Medicare patients. For example, primary Medicare patients are older and have higher comorbidity score [22]. There was limited data availability on laboratory results (40% of patients in Clinformatics^®^ and only 10% of patients in MarketScan^®^). The onset of T2D diagnosis was defined as we used claim codes to identify patients with T2D, not fasting blood sugar or HbA1c levels. Race was only available from the Clinformatics^®^ database but not from IBM MarketScan^®^. Fourthly, because the data were extracted from insurance claims, we did not have information on donor characteristics, cross matching, induction regimen, type of rejection, or treatment of rejection. Fifthly, obesity-related diagnoses are generally under-coded, and direct BMI measurement is not available from claims databases; however, our obesity-related codes were validated and had a high specificity (99.7% specificity for obesity, and 97.4% specificity for overweight) [21]. Lastly, KTRs are at increased risk of developing post-transplant diabetes, and we were unable to distinguish patients who developed T2D post-transplant from patients who had pre-transplant diabetes. Regarding the effectiveness and safety of SGLT2i and GLP1RA therapy in KTRs with T2D, a propensity-score-matched comparative study by our research team is currently underway.

## 5. Conclusions

In KTRs with T2D, the initiation of SGLT2i and GLP1RA increased over time. Insulin had the highest utilization among all GLMs, but its use declined over time. SGLT2is were initiated more frequently in patients with higher CV comorbidities and proteinuria, while GLP1RAs were initiated more frequently in patients with obesity, compared to insulin initiators. Future studies are needed on the effectiveness and safety of SGLT2i and GLP1RA among KTRs with T2D.

## Figures and Tables

**Table 1:** Clinical characteristics of all KTRs with treated T2D initiated on SGLT2i, GLP1RA, and insulin.

Clinical Characteristic	All KTRs with Treated T2D	SGLT2i Initiators	GLP1RA Initiators	Insulin Initiators
**Number of patients**	33,913	1009	2149	13,641
Mean age (SD)	59.33 (10.95)	61.06 (10.34)	58.00 (10.01)	58.64 (10.65)
Male; *n* (%)	21,055 (62.1%)	654 (64.8%)	1213 (56.4%)	8731 (64.0%)
**Race categories** *				
White; *n* (%)	8958 (48.6%)	360 (46.5%)	658 (43.7%)	658 (43.7%)
Black; *n* (%)	3884 (21.1%)	150 (19.4%)	332 (22.0%)	332 (22.0%)
Asian; *n* (%)	965 (5.2%)	39 (5.0%)	62 (4.1%)	62 (4.1%)
Hispanic; *n* (%)	2934 (15.9%)	129 (16.6%)	262 (17.4%)	262 (17.4%)
Other or unknown; *n* (%)	1678 (9.1%)	97 (12.5%)	193 (12.8%)	193 (12.8%)
**Combined comorbidity score**				
mean (SD)	5.33 (3.10)	5.41 (3.25)	5.04 (2.79)	5.82 (3.16)
**Frailty Score**				
0.00–0.14 (robust); *n* (%)	7272 (21.4%)	224 (22.2%)	500 (23.3%)	2426 (17.8%)
0.15–0.24 (pre-frail); *n* (%)	20,855 (61.5%)	633 (62.7%)	1354 (63.0%)	8644 (63.4%)
≥0.25 (frail); *n* (%)	5786 (17.1%)	152 (15.1%)	295 (13.7%)	2571 (18.8%)
**Diabetes characteristics**				
Diabetic neuropathy; *n* (%)	12,035 (35.5%)	359 (35.6%)	845 (39.3%)	5499 (40.3%)
Diabetic retinopathy; *n* (%)	10,673 (31.5%)	246 (24.4%)	708 (32.9%)	5026 (36.8%)
Diabetic ketoacidosis; *n* (%)	1142 (3.4%)	12 (1.2%)	42 (2.0%)	644 (4.7%)
Hypoglycemia; *n* (%)	3508 (10.3%)	79 (7.8%)	212 (9.9%)	1763 (12.9%)
Mean HbA1c (SD) **	7.37 (1.57)	7.54 (1.59)	7.56 (1.63)	7.66 (1.74)
**Cardiovascular comorbidities**				
Hypertension; *n* (%)	30,893 (91.1%)	948 (94.0%)	1989 (92.6%)	12,630 (92.6%)
Hyperlipidemia; *n* (%)	26,013 (76.7%)	855 (84.7%)	1800 (83.8%)	10,631 (77.9%)
Coronary atherosclerosis; *n* (%)	11,632 (34.3%)	371 (36.8%)	656 (30.5%)	4977 (36.5%)
Congestive heart failure; *n* (%)	8367 (24.7%)	300 (29.7%)	512 (23.8%)	3681 (27.0%)
Atrial fibrillation; *n* (%)	4568 (13.5%)	186 (18.4%)	239 (11.1%)	2002 (14.7%)
Ischemic stroke; *n* (%)	4070 (12.0%)	132 (13.1%)	181 (8.4%)	1709 (12.5%)
Peripheral arterial disease; *n* (%)	6155 (18.1%)	172 (17.0%)	362 (16.8%)	2742 (20.1%)
**Metabolic comorbidities**				
Obesity ^†^; *n* (%)	10,978 (32.4%)	421 (41.7%)	1141 (53.1%)	4673 (34.3%)
Overweight ^‡^; *n* (%)	3279 (9.7%)	121 (12.0%)	249 (11.6%)	1547 (11.3%)
**Renal comorbidities**				
Proteinuria; *n* (%)	5918 (17.5%)	252 (25.0%)	389 (18.1%)	2383 (17.5%)
Urinary tract infection; *n* (%)	8117 (23.9%)	224 (22.2%)	565 (26.3%)	3552 (26.0%)
Genital mycotic infection; *n* (%)	746 (2.2%)	32 (3.2%)	71 (3.3%)	333 (2.4%)
Hyperkalemia; *n* (%)	7153 (21.1%)	172 (17.0%)	367 (17.1%)	3411 (25.0%)
Acute kidney injury; *n* (%)	10,147 (29.9%)	283 (28.0%)	572 (26.6%)	4826 (35.4%)
Mean creatinine (SD, mg/dL) **	2.29 (2.06)	1.45 (0.84)	1.93 (1.76)	2.38 (2.14)

Note: Percentages were calculated with the total sample size as the denominator. GLP1RA, glucagon-like peptide-1 receptor agonists; SD, standard deviation; SGLT2i, sodium glucose cotransporter-2 inhibitors. * Race data only available in Clinformatics^®^. ** Laboratory data not available in all patients. Mean and standard deviations calculated from patients with available laboratory data. ^†^ defined as BMI ≥ 30.0 kg/m^2^. ^‡^ defined as BMI 25.0–29.9 kg/m^2^.

## Data Availability

Collected data relevant to the results of this study can be found in the Supplementary Materials. Raw data cannot be shared due to the agreements with database carriers.

## Supplementary Materials

The following supporting information can be downloaded at: https://www.mdpi.com/article/10.3390/jcm14020651/s1, Table S1: Clinical characteristics of KTR with treated T2D, patients initiated on SGLT2i, GLP1RA, and Insulin from pooled databases; Table S2: Clinical characteristics of KTR with treated T2D from Optum’s de-identified Clinformatics^®^ Data Mart Database, MarketScan^®^ and pooled databases, 2014–2023; Figure S1. Trends of glucose-lowering medication use (any use) from Optum’s de-identified Clinformatics^®^ Data Mart Database, 2014–2023; Figure S2: Trends of glucose-lowering medication initiation (incident use) from Optum’s de-identified Clinformatics^®^ Data Mart Database, 2014–2023; Figure S3: Trends of glucose-lowering medication use (any use) from MarketScan^®^ database, 2014–2021; Figure S4: Trends of glucose-lowering medication initiation (incident use) from MarketScan^®^ database, 2014–2021.

## Abbreviations

The following abbreviations are used in this manuscript:

AKI	acute kidney injury
BMI	body mass index
CKD	chronic kidney disease
CV	cardiovascular
DPP4i	dipeptidyl peptidase-4 inhibitors
GLM	glucose-lowering medications
GLP1RA	glucagon-like peptide-1 receptor agonists
ICD	International Classification of Diseases
KTR	kidney transplant recipients
RCT	randomized controlled trials
SGLT2i	sodium glucose transporter-2 inhibitors
T2D	type 2 diabetes
TZD	thiazolidinediones
UTI	urinary tract infection
