# Supplementary material for: Utilization Trends of Glucose-Lowering Medications Among Adult Kidney Transplant Recipients with Type 2 Diabetes in the United States
Source: J Clin Med. 2025 Jan 20;14(2):651. doi: 10.3390/jcm14020651 (PMC11766230; doi:10.3390/jcm14020651)
Supplement: Supplementary file 1 [file jcm-14-00651-s001.zip › jcm-3424406-supplementary.pdf]

Supplementary Materials

Table of Contents

Table S1. Clinical characteristics of KTR with treated T2D, patients initiated on SGLT2i, GLP1RA, and Insulin from pooled databases. **Page 2**

Table S2. Clinical characteristics of KTR with treated T2D from Optum's de-identified Clinformatics® Data Mart Database, MarketScan® and pooled databases, 2014-2023. **Page 5**

Figure S1. Trends of glucose-lowering medication use (any use) from Optum's de-identified Clinformatics® Data Mart Database, 2014-2023. **Page 8**

FigureS2. Trends of glucose-lowering medication initiation (incident use) from Optum's de-identified Clinformatics® Data Mart Database, 2014-2023. **Page 9**

Figure S3. Trends of glucose-lowering medication use (any use) from MarketScan® database, 2014-2021. **Page 10**

Figure S4. Trends of glucose-lowering medication initiation (incident use) from MarketScan® database, 2014-2021. **Page 11**

Table S1. Clinical characteristics of KTR with treated T2D, patients initiated on SGLT2i, GLP1RA, and Insulin from pooled databases.

Clinical Characteristics	All patients	SGLT2i	GLP1RA	Insulin
Number of patients	33,913	1,009	2,149	13,641
Age				
...mean (SD)	59.33 (10.95)	61.06 (10.34)	58.00 (10.01)	58.64 (10.65)
Male; n (%)	21,055 (62.1%)	654 (64.8%)	1,213 (56.4%)	8,731 (64.0%)
Geographic region				
...Northeast; n (%)	4,713 (13.9%)	109 (10.8%)	283 (13.2%)	1,793 (13.1%)
...South; n (%)	16,173 (47.7%)	546 (54.1%)	1,148 (53.4%)	6,886 (50.5%)
...Midwest; n (%)	7,422 (21.9%)	188 (18.6%)	412 (19.2%)	2,784 (20.4%)
...West; n (%)	5,605 (16.5%)	166 (16.5%)	306 (14.2%)	2,178 (16.0%)
*Race categories**				
...White; n (%)	8,958 (48.6%)	360 (46.5%)	658 (43.7%)	658 (43.7%)
...Black; n (%)	3,884 (21.1%)	150 (19.4%)	332 (22.0%)	332 (22.0%)
...Asian; n (%)	965 (5.2%)	39 (5.0%)	62 (4.1%)	62 (4.1%)
...Hispanic; n (%)	2,934 (15.9%)	129 (16.6%)	262 (17.4%)	262 (17.4%)
...Other or unknown; n (%)	1,678 (9.1%)	97 (12.5%)	193 (12.8%)	193 (12.8%)
Combined comorbidity score				
...mean (SD)	5.33 (3.10)	5.41 (3.25)	5.04 (2.79)	5.82 (3.16)
Frailty Score				
...0.00 - 0.14 (robust); n (%)	7,272 (21.4%)	224 (22.2%)	500 (23.3%)	2,426 (17.8%)
...0.15 - 0.24 (pre-frail); n (%)	20,855 (61.5%)	633 (62.7%)	1,354 (63.0%)	8,644 (63.4%)
...≥ 0.25 (frail); n (%)	5,786 (17.1%)	152 (15.1%)	295 (13.7%)	2,571 (18.8%)
Diabetes characteristics				
Diabetes with peripheral circulatory disorders; n (%)	1,746 (5.1%)	15 (1.5%)	43 (2.0%)	684 (5.0%)
Diabetic foot; n (%)	3,448 (10.2%)	99 (9.8%)	222 (10.3%)	1,594 (11.7%)
Diabetic neuropathy; n (%)	12,035 (35.5%)	359 (35.6%)	845 (39.3%)	5,499 (40.3%)
Diabetic retinopathy; n (%)	10,673 (31.5%)	246 (24.4%)	708 (32.9%)	5,026 (36.8%)
Diabetes with other ophthalmic manifestations; n (%)	4,743 (14.0%)	92 (9.1%)	234 (10.9%)	1,764 (12.9%)
Diabetic ketoacidosis; n (%)	1,142 (3.4%)	12 (1.2%)	42 (2.0%)	644 (4.7%)
Hyperosmolar hyperglycemic nonketotic syndrome; n (%)	585 (1.7%)	13 (1.3%)	41 (1.9%)	339 (2.5%)
Hypoglycemia; n (%)	3,508 (10.3%)	79 (7.8%)	212 (9.9%)	1,763 (12.9%)
Lower-limb amputations; n (%)	1,771 (5.2%)	47 (4.7%)	112 (5.2%)	933 (6.8%)
HbA1c (%)**				
...mean (SD)	7.37 (1.57)	7.54 (1.59)	7.56 (1.63)	7.66 (1.74)
Cardiovascular comorbidities				
Hypertension; n (%)	30,893 (91.1%)	948 (94.0%)	1,989 (92.6%)	12,630 (92.6%)
Hyperlipidemia; n (%)	26,013 (76.7%)	855 (84.7%)	1,800 (83.8%)	10,631 (77.9%)
Coronary atherosclerosis; n (%)	11,632 (34.3%)	371 (36.8%)	656 (30.5%)	4,977 (36.5%)
Congestive heart failure; n (%)	8,367 (24.7%)	300 (29.7%)	512 (23.8%)	3,681 (27.0%)
Atrial fibrillation; n (%)	4,568 (13.5%)	186 (18.4%)	239 (11.1%)	2,002 (14.7%)
Ischemic stroke; n (%)	4,070 (12.0%)	132 (13.1%)	181 (8.4%)	1,709 (12.5%)
Peripheral arterial disease; n (%)	6,155 (18.1%)	172 (17.0%)	362 (16.8%)	2,742 (20.1%)
Smoking; n (%)	7,218 (21.3%)	283 (28.0%)	518 (24.1%)	3,251 (23.8%)

Metabolic comorbidities				
Obesity†; n (%)	10,978 (32.4%)	421 (41.7%)	1,141 (53.1%)	4,673 (34.3%)
Overweight‡; n (%)	3,279 (9.7%)	121 (12.0%)	249 (11.6%)	1,547 (11.3%)
Renal comorbidities				
Proteinuria; n (%)	5,918 (17.5%)	252 (25.0%)	389 (18.1%)	2,383 (17.5%)
Urinary tract infection; n (%)	8,117 (23.9%)	224 (22.2%)	565 (26.3%)	3,552 (26.0%)
Genital mycotic infection; n (%)	746 (2.2%)	32 (3.2%)	71 (3.3%)	333 (2.4%)
Hyperkalemia; n (%)	7,153 (21.1%)	172 (17.0%)	367 (17.1%)	3,411 (25.0%)
Acute kidney injury; n (%)	10,147 (29.9%)	283 (28.0%)	572 (26.6%)	4,826 (35.4%)
Creatinine (mg/dL)**				
...mean (SD)	2.29 (2.06)	1.45 (0.84)	1.93 (1.76)	2.38 (2.14)
Endocrine comorbidities				
Fractures; n (%)	1,315 (3.9%)	35 (3.5%)	81 (3.8%)	573 (4.2%)
Hypothyroidism; n (%)	6,278 (18.5%)	220 (21.8%)	438 (20.4%)	2,520 (18.5%)
Hyperthyroidism; n (%)	3,074 (9.1%)	106 (10.5%)	244 (11.4%)	1,284 (9.4%)
Other comorbidities				
Cancer; n (%)	3,414 (10.1%)	98 (9.7%)	176 (8.2%)	1,347 (9.9%)
Asthma; n (%)	2,627 (7.7%)	82 (8.1%)	177 (8.2%)	1,084 (7.9%)
COPD; n (%)	3,374 (9.9%)	113 (11.2%)	176 (8.2%)	1,371 (10.1%)
Liver disease; n (%)	6,082 (17.9%)	171 (16.9%)	308 (14.3%)	2,727 (20.0%)
Alcohol abuse or dependence; n (%)	841 (2.5%)	25 (2.5%)	37 (1.7%)	429 (3.1%)
Glucose-lowering medications				
SGLT2i; n (%)	2,735 (8.1%)	-	1,451 (67.5%)	12,589 (92.3%)
GLP1RA; n (%)	3,902 (11.5%)	328 (32.5%)	-	927 (6.8%)
Insulins; n (%)	25,433 (75.0%)	199 (19.7%)	343 (16.0%)	-
Metformin; n (%)	7,404 (21.8%)	069 (6.8%)	119 (5.5%)	205 (1.5%)
Sulfonylureas†; n (%)	7,428 (21.9%)	859 (85.1%)	374 (17.4%)	285 (2.1%)
DPP4i; n (%)	5,454 (16.1%)	189 (18.7%)	1,347 (62.7%)	852 (6.2%)
Thiazolidinediones; n (%)	1,315 (3.9%)	149 (14.8%)	164 (7.6%)	923 (6.8%)
Miscellaneous diabetes medications‡; n (%)	1,075 (3.2%)	23 (2.3%)	54 (2.5%)	144 (1.1%)
Number of glucose-lowering medications				
...mean (SD)	1.21 (0.51)	1.82 (0.94)	1.69 (0.80)	1.25 (0.54)
Immunosuppressive agents				
Calcineurin inhibitors (cyclosporine/tacrolimus); n (%)	25,073 (73.9%)	700 (69.4%)	1,666 (77.5%)	11,155 (81.8%)
Belatacept; n (%)	238 (0.7%)	19 (1.9%)	18 (0.8%)	116 (0.9%)
Mycophenolate mofetil/Mycophenolic acid; n (%)	22,670 (66.8%)	609 (60.4%)	1,495 (69.6%)	10,108 (74.1%)
Mammalian target of rapamycin (mTOR) inhibitors; n (%)	3,056 (9.0%)	82 (8.1%)	131 (6.1%)	1,132 (8.3%)
Azathioprine; n (%)	1,252 (3.7%)	47 (4.7%)	68 (3.2%)	807 (5.9%)
Oral corticosteroids; n (%)	20,041 (59.1%)	605 (60.0%)	1,314 (61.1%)	9,092 (66.7%)
Anti-microbial agents				
Trimethoprim/sulfamethoxazole; n (%)	20,277 (59.8%)	437 (43.3%)	1,013 (47.1%)	7,872 (57.7%)
Atovaquone; n (%)	1,279 (3.8%)	32 (3.2%)	70 (3.3%)	590 (4.3%)
Diuretics and binders				
Loop diuretics; n (%)	13,896 (41.0%)	374 (37.1%)	748 (34.8%)	5,692 (41.7%)
Thiazide and thiazide-like diuretics; n (%)	3,621 (10.7%)	128 (12.7%)	235 (10.9%)	1,317 (9.7%)
Mineralocorticoid receptor antagonists; n (%)	1,996 (5.9%)	114 (11.3%)	114 (5.3%)	787 (5.8%)

Potassium binders; n (%)	2,128 (6.3%)	55 (5.5%)	107 (5.0%)	988 (7.2%)
Cardiovascular medications				
ACEI/ARB/ARNI; n (%)	17,673 (52.1%)	634 (62.8%)	1,098 (51.1%)	6,523 (47.8%)
Beta blockers; n (%)	23,458 (69.2%)	675 (66.9%)	1,409 (65.6%)	9,628 (70.6%)
Calcium channel blockers; n (%)	19,885 (58.6%)	533 (52.8%)	1,196 (55.7%)	8,214 (60.2%)
Antiarrhythmics; n (%)	1,026 (3.0%)	52 (5.2%)	55 (2.6%)	432 (3.2%)
Nitrates and other antianginal agents; n (%)	3,330 (9.8%)	111 (11.0%)	162 (7.5%)	1,284 (9.4%)
Statins; n (%)	24,142 (71.2%)	771 (76.4%)	1,681 (78.2%)	9,500 (69.6%)
Antiplatelet agents; n (%)	6,284 (18.5%)	180 (17.8%)	331 (15.4%)	2,602 (19.1%)
Anticoagulants (oral); n (%)	4,477 (13.2%)	167 (16.6%)	264 (12.3%)	1,938 (14.2%)
Other medications				
COPD & asthma medications; n (%)	4,784 (14.1%)	149 (14.8%)	310 (14.4%)	1,834 (13.4%)
Opioids; n (%)	17,360 (51.2%)	403 (39.9%)	929 (43.2%)	7,205 (52.8%)
NSAIDs; n (%)	4,612 (13.6%)	165 (16.4%)	309 (14.4%)	1,765 (12.9%)

Note: Percentages were calculated with the total sample size as the denominator. ACEI, angiotensin-converting enzyme inhibitors; ARB, angiotensin receptor blockers; ARNI, angiotensin receptor/neprilysin inhibitors; COPD, chronic obstructive pulmonary disease; NSAIDs; non-steroidal anti-inflammatory drugs; SD, standard deviation.
*race data only available in Optum's de-identified Clinformatics® Data Mart Database.
**laboratory data not available in all patients. Mean and standard deviation calculated from patients with laboratory data available
†defined as BMI ≥ 30.0 kg/m^2
‡defined as BMI 25.0-29.9 kg/m^2

Table S2. Clinical characteristics of KTR with treated T2D from Optum's de-identified Clinformatics® Data Mart Database, MarketScan® and pooled databases, 2014-2023.

Clinical Characteristics	Clinformatics®	MarketScan®	Pooled
Number of patients	18,419	15,494	33,913
Age			
...mean (SD)	61.16 (11.15)	57.15 (10.70)	59.33 (10.95)
Male; n (%)	11,242 (61.0%)	9,813 (63.3%)	21,055 (62.1%)
Geographic region			
...Northeast; n (%)	1,911 (10.4%)	2,802 (18.1%)	4,713 (13.9%)
...South; n (%)	9,542 (51.8%)	6,631 (42.8%)	16,173 (47.7%)
...Midwest; n (%)	3,555 (19.3%)	3,867 (25.0%)	7,422 (21.9%)
...West; n (%)	3,411 (18.5%)	2,194 (14.2%)	5,605 (16.5%)
*Race categories**			
...White; n (%)	8,958 (48.6%)	-	8,958 (48.6%)
...Black; n (%)	3,884 (21.1%)	-	3,884 (21.1%)
...Asian; n (%)	965 (5.2%)	-	965 (5.2%)
...Hispanic; n (%)	2,934 (15.9%)	-	2,934 (15.9%)
...Other or unknown; n (%)	1,678 (9.1%)	-	1,678 (9.1%)
Combined comorbidity score			
...mean (SD)	5.95 (3.30)	4.59 (2.85)	5.33 (3.10)
Frailty Score			
...0.00 - 0.14 (robust); n (%)	3,171 (17.2%)	4,101 (26.5%)	7,272 (21.4%)
...0.15 - 0.24 (pre-frail); n (%)	11,286 (61.3%)	9,569 (61.8%)	20,855 (61.5%)
... ≥ 0.25 (frail); n (%)	3,962 (21.5%)	1,824 (11.8%)	5,786 (17.1%)
Diabetes characteristics			
Diabetes with peripheral circulatory disorders; n (%)	884 (4.8%)	862 (5.6%)	1,746 (5.1%)
Diabetic ketoacidosis; n (%)	646 (3.5%)	496 (3.2%)	1,142 (3.4%)
Diabetic foot; n (%)	2,070 (11.2%)	1,378 (8.9%)	3,448 (10.2%)
Diabetic neuropathy; n (%)	7,622 (41.4%)	4,413 (28.5%)	12,035 (35.5%)
Diabetic retinopathy; n (%)	6,467 (35.1%)	4,206 (27.1%)	10,673 (31.5%)
Diabetes with other ophthalmic manifestations; n (%)	2,066 (11.2%)	2,677 (17.3%)	4,743 (14.0%)
Hyperosmolar hyperglycemic nonketotic syndrome; n (%)	358 (1.9%)	227 (1.5%)	585 (1.7%)
Hypoglycemia; n (%)	2,381 (12.9%)	1,127 (7.3%)	3,508 (10.3%)
Lower-limb amputations; n (%)	1,279 (6.9%)	492 (3.2%)	1,771 (5.2%)
HbA1c (%)**			
...mean (SD)	7.40 (1.56)	7.33 (1.59)	7.37 (1.57)
Cardiovascular comorbidities			
Hypertension; n (%)	17,388 (94.4%)	13,505 (87.2%)	30,893 (91.1%)
Hyperlipidemia; n (%)	15,474 (84.0%)	10,539 (68.0%)	26,013 (76.7%)
Coronary atherosclerosis; n (%)	7,101 (38.6%)	4,531 (29.2%)	11,632 (34.3%)
Congestive heart failure; n (%)	5,402 (29.3%)	2,965 (19.1%)	8,367 (24.7%)

Atrial fibrillation; n (%)	2,900 (15.7%)	1,668 (10.8%)	4,568 (13.5%)
Ischemic stroke; n (%)	2,487 (13.5%)	1,583 (10.2%)	4,070 (12.0%)
Peripheral arterial disease; n (%)	3,962 (21.5%)	2,193 (14.2%)	6,155 (18.1%)
Smoking; n (%)	5,683 (30.9%)	1,535 (9.9%)	7,218 (21.3%)
Metabolic comorbidities			
Obesity[§]; n (%)	7,110 (38.6%)	3,868 (25.0%)	10,978 (32.4%)
Overweight[¶]; n (%)	2,428 (13.2%)	851 (5.5%)	3,279 (9.7%)
Renal comorbidities			
Proteinuria; n (%)	3,731 (20.3%)	2,187 (14.1%)	5,918 (17.5%)
Urinary tract infection; n (%)	4,962 (26.9%)	3,155 (20.4%)	8,117 (23.9%)
Hyperkalemia; n (%)	4,627 (25.1%)	2,526 (16.3%)	7,153 (21.1%)
Acute kidney injury; n (%)	5,979 (32.5%)	4,168 (26.9%)	10,147 (29.9%)
Creatinine (mg/dL)**			
...mean (SD)	2.40 (2.12)	2.17 (1.99)	2.29 (2.06)
Endocrine comorbidities			
Fractures; n (%)	834 (4.5%)	481 (3.1%)	1,315 (3.9%)
Hypothyroidism; n (%)	3,844 (20.9%)	2,434 (15.7%)	6,278 (18.5%)
Hyperthyroidism; n (%)	1,817 (9.9%)	1,257 (8.1%)	3,074 (9.1%)
Other medical comorbidities			
Cancer; n (%)	2,320 (12.6%)	1,094 (7.1%)	3,414 (10.1%)
Asthma; n (%)	1,729 (9.4%)	898 (5.8%)	2,627 (7.7%)
COPD; n (%)	2,329 (12.6%)	1,045 (6.7%)	3,374 (9.9%)
Liver disease; n (%)	3,642 (19.8%)	2,440 (15.7%)	6,082 (17.9%)
Alcohol abuse or dependence; n (%)	557 (3.0%)	284 (1.8%)	841 (2.5%)
Glucose-lowering medications			
SGLT2i; n (%)	2,040 (11.1%)	695 (4.5%)	2,735 (8.1%)
GLP1RA; n (%)	2,002 (10.9%)	1,900 (12.3%)	3,902 (11.5%)
Insulins; n (%)	13,831 (75.1%)	11,602 (74.9%)	25,433 (75.0%)
Metformin; n (%)	4,044 (22.0%)	3,360 (21.7%)	7,404 (21.8%)
Sulfonylureas[†]; n (%)	3,896 (21.2%)	3,532 (22.8%)	7,428 (21.9%)
DPP4i; n (%)	2,859 (15.5%)	2,595 (16.7%)	5,454 (16.1%)
Thiazolidinediones; n (%)	731 (4.0%)	584 (3.8%)	1,315 (3.9%)
Miscellaneous diabetes medications[‡]; n (%)	472 (2.6%)	603 (3.9%)	1,075 (3.2%)
Number of glucose-lowering medications	13,831 (75.1%)	11,602 (74.9%)	25,433 (75.0%)
...mean (SD)	1.23 (0.52)	1.19 (0.49)	1.21 (0.51)
Other medications			
Immunosuppressive agents			
Calcineurin inhibitors (cyclosporine/tacrolimus); n (%)	13,381 (72.6%)	11,692 (75.5%)	25,073 (73.9%)
Belatacept; n (%)	214 (1.2%)	24 (0.2%)	238 (0.7%)
Mycophenolate mofetil/Mycophenolic acid; n (%)	11,897 (64.6%)	10,773 (69.5%)	22,670 (66.8%)
Mammalian target of rapamycin (mTOR) inhibitors; n (%)	1,334 (7.2%)	1,722 (11.1%)	3,056 (9.0%)
Azathioprine; n (%)	654 (3.6%)	598 (3.9%)	1,252 (3.7%)
Oral corticosteroids; n (%)	10,867 (59.0%)	9,174 (59.2%)	20,041 (59.1%)
Anti-microbial agents			
Trimethoprim-sulfamethoxazole; n (%)	10,253 (55.7%)	10,024 (64.7%)	20,277 (59.8%)
Atovaquone; n (%)	662 (3.6%)	617 (4.0%)	1,279 (3.8%)

Diuretics and binders			
Loop diuretics; n (%)	7,721 (41.9%)	6,175 (39.9%)	13,896 (41.0%)
Thiazide and thiazide-like diuretics; n (%)	1,993 (10.8%)	1,628 (10.5%)	3,621 (10.7%)
Mineralocorticoid receptor antagonists; n (%)	1,103 (6.0%)	893 (5.8%)	1,996 (5.9%)
Potassium binders; n (%)	1,226 (6.7%)	902 (5.8%)	2,128 (6.3%)
Cardiovascular medications			
ACEI/ARB/ARNI; n (%)	9,458 (51.3%)	8,215 (53.0%)	17,673 (52.1%)
Beta blockers; n (%)	12,719 (69.1%)	10,739 (69.3%)	23,458 (69.2%)
Calcium channel blockers; n (%)	10,933 (59.4%)	8,952 (57.8%)	19,885 (58.6%)
Digoxin; n (%)	211 (1.1%)	163 (1.1%)	374 (1.1%)
Antiarrhythmics; n (%)	583 (3.2%)	443 (2.9%)	1,026 (3.0%)
Nitrates and other antianginal agents; n (%)	1,903 (10.3%)	1,427 (9.2%)	3,330 (9.8%)
Statins; n (%)	13,509 (73.3%)	10,633 (68.6%)	24,142 (71.2%)
Antiplatelet agents; n (%)	3,330 (18.1%)	2,954 (19.1%)	6,284 (18.5%)
Anticoagulants (oral); n (%)	2,698 (14.6%)	1,779 (11.5%)	4,477 (13.2%)
Miscellaneous medications			
COPD & asthma medications; n (%)	2,719 (14.8%)	2,065 (13.3%)	4,784 (14.1%)
Opioids; n (%)	9,287 (50.4%)	8,073 (52.1%)	17,360 (51.2%)
NSAIDs; n (%)	2,353 (12.8%)	2,259 (14.6%)	4,612 (13.6%)

Note: Percentages were calculated with the total sample size as the denominator. ACEI, angiotensin-converting enzyme inhibitors; ARB, angiotensin receptor blockers; ARNI, angiotensin receptor/neprilysin inhibitors; COPD, chronic obstructive pulmonary disease; DPP4i, dipeptidyl peptidase-4 inhibitors; GLP1RA, glucagon-like peptide-1 receptor agonists; KTR, kidney transplant recipients; NSAIDs; non-steroidal anti-inflammatory drugs; SD, standard deviation; SGLT2i, sodium-glucose cotransporter-2 inhibitors; T2D, type 2 diabetes.

*race data only available in Optum's de-identified Clinformatics® Data Mart Database.

**laboratory data not available in all patients. Mean and standard deviation calculated from patients with laboratory data available.

†refers to second-generation sulfonylureas, including glyburide, gliclazide, glipizide, and glimepiride.

‡includes first generation sulfonylureas, α-glucosidase inhibitors, pramlintide, and meglitinide.

§defined as BMI ≥ 30.0 kg/m^2

¶defined as BMI 25.0-29.9 kg/m^2

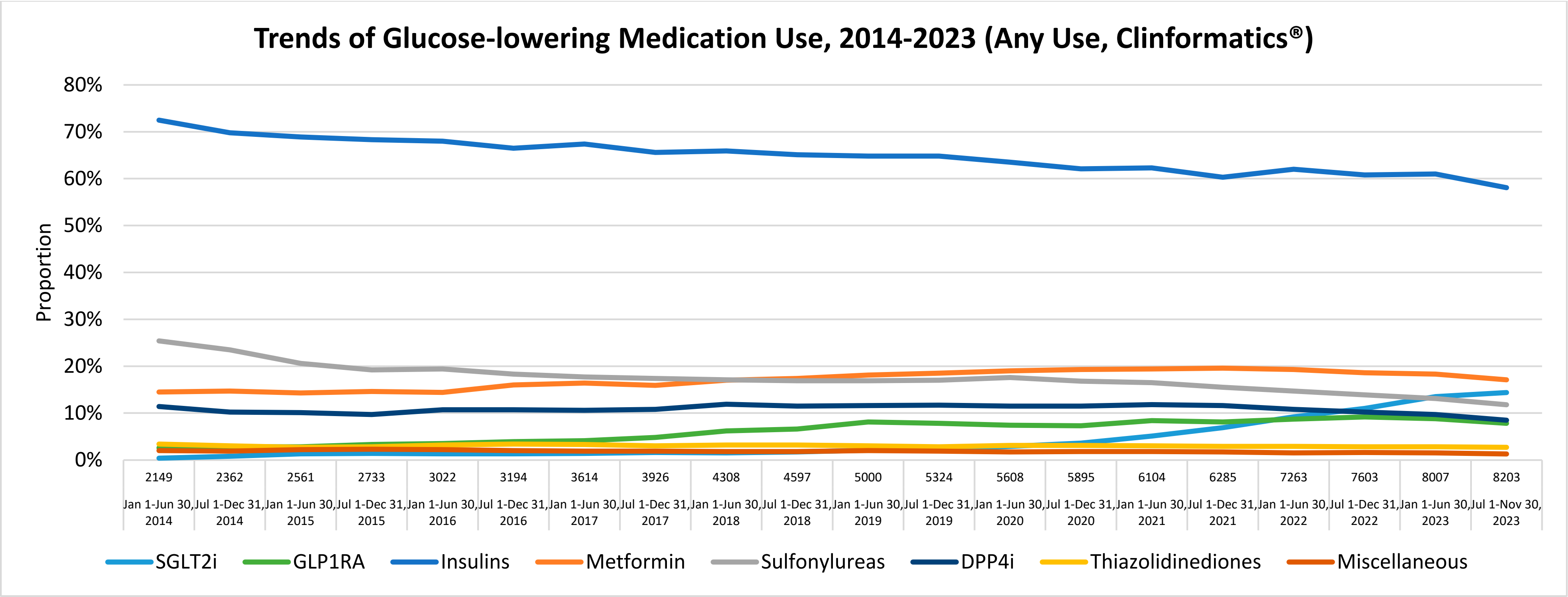

Figure S1. Trends of glucose-lowering medication use (any use) from Optum's de-identified Clinformatics® Data Mart Database (Clinformatics®), 2014-2023. Data range from January 1, 2014 to November 30, 2023. DPP4i, dipeptidyl peptidase-4 inhibitors; GLP1RA, glucagon-like peptide receptor agonists; SGLT2i, sodium glucose cotransporter-2 inhibitors.

Sulfonylureas refers to second-generation sulfonylureas, including glyburide, gliclazide, glipizide, and glimepiride.

Miscellaneous includes first generation sulfonylureas, α-glucosidase inhibitors, pramlintide, and meglitinide.

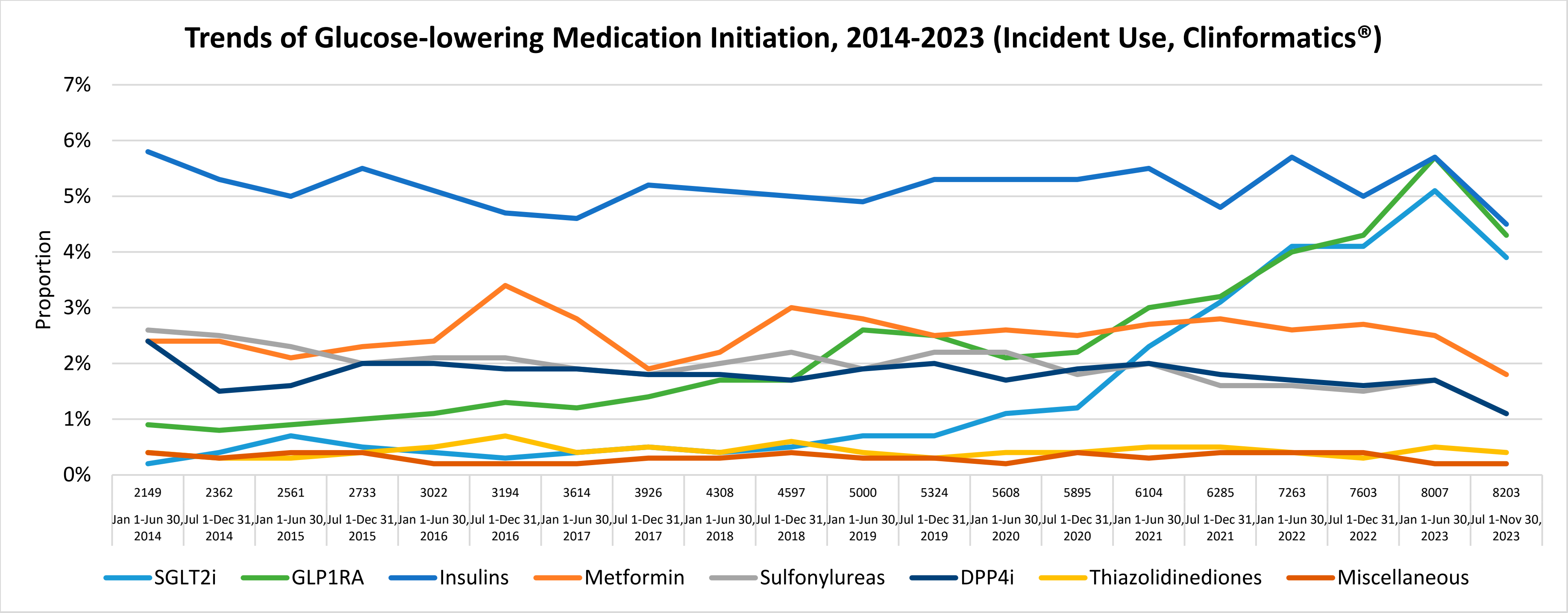

Figure S2. Trends of glucose-lowering medication initiation (incident use) from Optum's de-identified Clinformatics® Data Mart Database (Clinformatics®), 2014-2023. Data range from January 1, 2014 to November 30, 2023. DPP4i, dipeptidyl peptidase-4 inhibitors; GLP1RA, glucagon-like peptide receptor agonists; SGLT2i, sodium glucose cotransporter-2 inhibitors.

Sulfonylureas refers to second-generation sulfonylureas, including glyburide, gliclazide, glipizide, and glimepiride.

Miscellaneous includes first generation sulfonylureas, α-glucosidase inhibitors, pramlintide, and meglitinide.

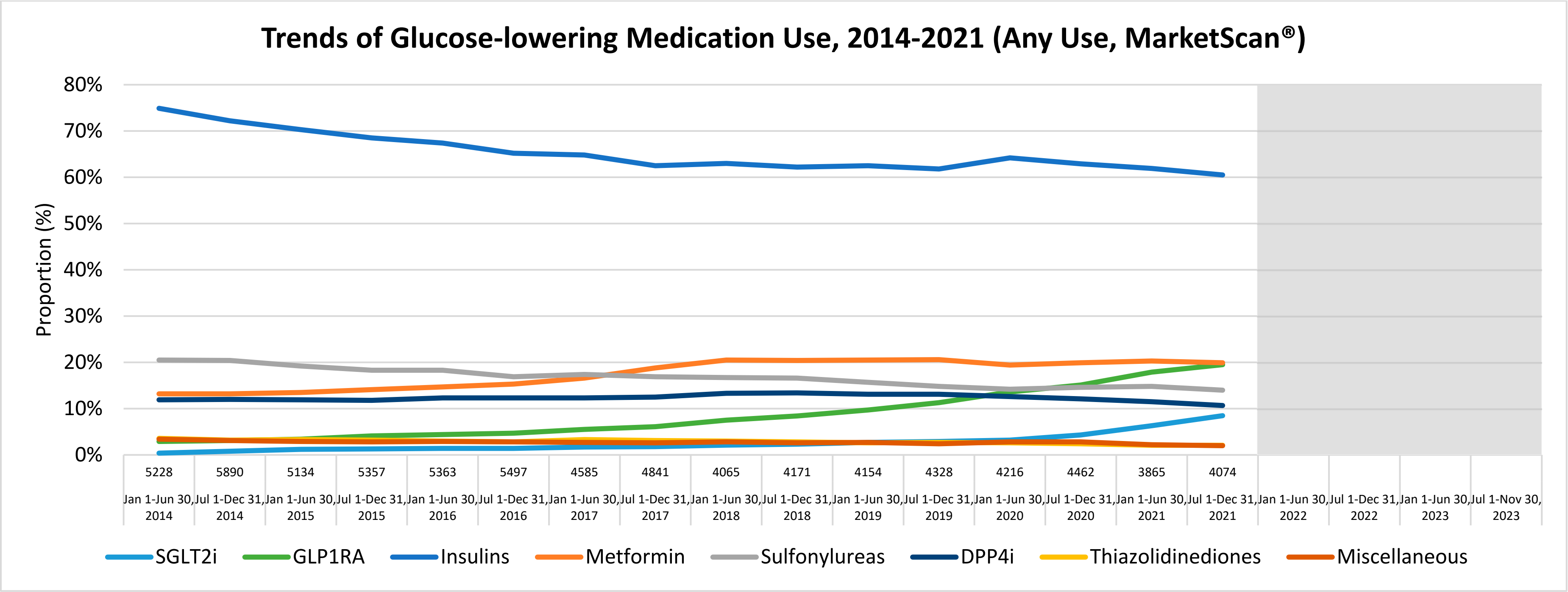

Figure S3. Trends of glucose-lowering medication use (any use) from MarketScan® database, 2014-2021. Data range from January 1, 2014 to December 31, 2021. DPP4i, dipeptidyl peptidase-4 inhibitors; GLP1RA, glucagon-like peptide receptor agonists; SGLT2i, sodium glucose cotransporter-2 inhibitors.
Sulfonylureas refers to second-generation sulfonylureas, including glyburide, gliclazide, glipizide, and glimepiride.
Miscellaneous includes first generation sulfonylureas, α-glucosidase inhibitors, pramlintide, and meglitinide.

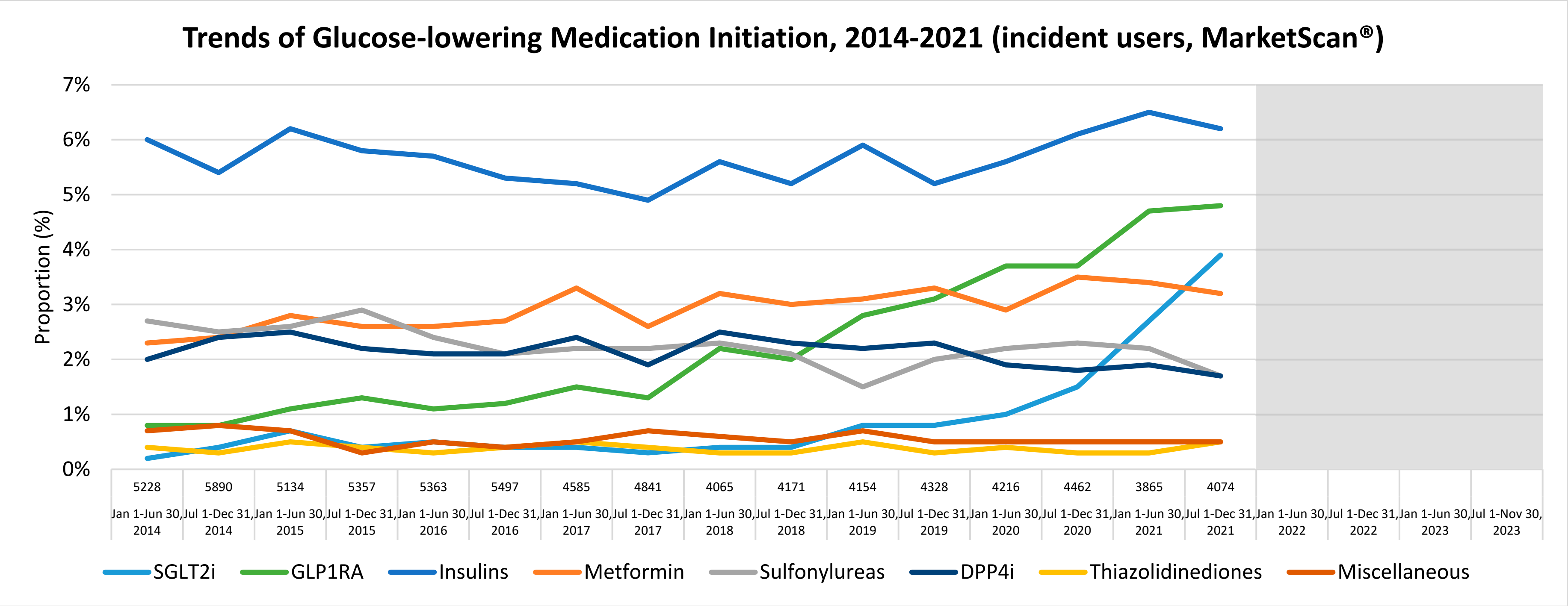

Figure S4. Trends of glucose-lowering medication initiation (incident use) from MarketScan® database, 2014-2021. Data range from January 1, 2014 to December 31, 2021. DPP4i, dipeptidyl peptidase-4 inhibitors; GLP1RA, glucagon-like peptide receptor agonists; SGLT2i, sodium glucose cotransporter-2 inhibitors. Sulfonylureas refers to second-generation sulfonylureas, including glyburide, gliclazide, glipizide, and glimepiride.
Miscellaneous includes first generation sulfonylureas, α-glucosidase inhibitors, pramlintide, and meglitinide.
